# Lavigne-Robichaud et al: Job strain and ischemic heart disease: the balance of methodological bias and implications for prevention. Author reply.

**DOI:** 10.5271/sjweh.4324

**Published:** 2026-09-01

**Authors:** Jens Peter Bonde, Stinna Skaaby, Esben M Flachs, Maureen Dollard, Katherine Keyes, Annika Rosengren, Ingrid AS Mehlum, Sigurd Mikkelsen

**Affiliations:** 1 Department of Occupational and Environmental Medicine, Bispebjerg and Frederiksberg University Hospital, Copenhagen, Denmark.; 2 Institute of Public Health, University of Copenhagen, Copenhagen, Denmark.; 3 Psychosocial Safety Climate Global Observatory, University of South Australia, Adelaide, Australia.; 4 Department of Epidemiology, Columbia University, New York, USA.; 5 Institute of Medicine, Sahlgrenska Academy, University of Gothenburg, Gothenburg, Sweden.

We appreciate Lavigne-Robichaud et al's critical remarks (1) to our systematic review addressing the epidemiological evidence for causal relations between job strain and the risk of ischemic heart disease (IHD) (2). Their main concern relates to the strength of the hypothesized association between job strain and IHD and, by extension, the magnitude of the population attributable fraction. They argue that our overall conclusion places too much emphasis on biases that inflate risk estimates and underrates biases that attenuate estimates towards the null. However, we believe it is premature to discuss in detail the strength of associations before a causal link is established with reasonable confidence.

The main objective of our review was to evaluate the epidemiological evidence that observed associations are indeed causal. We concluded that the current evidence neither substantiates nor excludes a causal association for several reasons, including, but not limited to, the presence of inflating or deflating biases (2).

That said, we appreciate the opportunity to discuss the sources of bias raised by Lavigne-Robichaud et al (1).

First, it is argued that dichotomizing exposure into "high" and "no-high" job strain deflates risk estimates because a subgroup of the no-high job strain group (passive jobs with low demands and low control) might also be associated with increased risk. However, others have found that results are broadly similar when different non-strain groups are used as reference (3). Thus, at present, the evidence for effects of passive jobs is not strong enough to deviate from the parsimonious use of "all others" as reference.

Second, the notion that the pooled risk estimate may be attenuated by sex because women develop IHD at older ages than men is supported by our review indicating slightly lower risk estimates among women. However, the confidence intervals substantially overlap [(2), table 4] and the small difference, if valid, may be due to several other factors and thus considered of minor importance.

Third, the use of job-exposure matrices (JEM) is *not* associated with attenuated risk estimates *if* the average exposure in the job groups is close to the true average (4). This condition is probably violated in most instances and may lead to attenuated risk estimates if the exposure misclassification is non-differential. However, misclassification may depend on potential confounders (eg, sex, socioeconomic status and psychosocial and lifestyle factors) in ways that inflate or deflate risk estimates. Note also that the JEM studies do not provide the independent assessment of exposure that is needed for stronger conclusions to be drawn. This is because most studies are based on JEM established by population-based questionnaire studies. We consider the lack of independent exposure assessment a major obstacle for causal inference.

Fourth, we agree that the prospective cohort study design in principle eliminates recall bias caused by later occurring manifest disease. But it is not obvious that subclinical or early stages of the disease cannot confound the association, potentially leading to inflated risk estimates even in prospective studies (3).

Fifth, self-reported psychosocial work exposures are inherently vulnerable to respondents' individual interpretations, particularly when they are asked to rate the intensity or frequency of subjective experiences. We acknowledge the references provided by Lavigne-Robichaud et al, but these studies do not rule out potential confounding by personality (5, 6). The influence of social context, attitudes, life experiences and personality traits remains unresolved and likely constitute bias regardless of study design (7).

We are aware that our review may be misused to discourage sound efforts to improve the psychosocial work environment. While this unfortunate risk is inherent to all critical appraisals of the literature, we believe it remains incumbent on researchers to provide the most evidence-based assessments possible so that we can collectively endeavor to improve science.
